# The impact of a global health elective on CanMEDS competencies and future practice

**DOI:** 10.1186/s12960-020-0447-4

**Published:** 2020-01-29

**Authors:** Ashley Lanys, Gena Krikler, Rachel F. Spitzer

**Affiliations:** 1 Faculty of Medicine, Department of Obstetrics and Gynaecology, University of Toronto, 123 Edward Street, Suite 1200, Toronto, Ontario M5G 1E2 Canada; 2 Department of Obstetrics and Gynaecology, Mount Sinai Hospital, Toronto, Ontario Canada; 3 Department of Obstetrics and Gynaecology, Women’s College Hospital, Toronto, Ontario Canada; 4 School of Medicine, Royal College of Surgeons in Ireland, Dublin, Ireland

**Keywords:** Global health, Reproductive health, CanMEDS competencies

## Abstract

**Background:**

There is evidence that participating in global health electives generates positive educational outcomes and personal benefits for medical trainees. The objective of this study was to examine the effect and impact that a global health elective has on CanMEDS competencies and anticipated future practice.

**Results:**

The medical expert, collaborator, leader, scholar, and professional CanMEDS competencies were self-perceived to be strongly impacted through this elective. A total of 94% of participants indicated it increased their strengths as a medical expert and leader, 82% indicated a major impact on the scholar competency, 88% of participants reported a strong impact as a professional, and 76% of participants indicated that it strongly impacted them as a collaborator. The majority of participants continue to have involvement in global health, and 88% of respondents found this elective to be influential on their current practice and beliefs.

**Conclusions:**

These results suggest that individuals who participated in this global health elective perceived value in their experience. These findings support our hypothesis that participation in this global health elective would generate self-perceived positive impacts. Global health electives may provide an opportunity for physicians to expand on their CanMEDS competencies and become more proficient in caring for diverse patient populations.

## Background

Living in an increasingly interdependent and interconnected world requires physicians to be proficient in global health in order to care for diverse patient populations. There is evidence that global health electives can generate positive educational outcomes and personal benefits for its participants. The Mayo Clinic reported enhanced experience with pathology, learning to work with limited resources, developing clinical and surgical skills, participating in resident education, and experiencing new population demographics as the acquired benefits to participating in their global health elective [1]. Similarly, the Boston Combined Residency Program found that global health electives significantly impacted clinical knowledge and skills, and systems awareness [2].

However, there is currently limited research that evaluates the impact global health electives have on the development of competencies within the CanMEDS framework.

The CanMEDS framework identifies and describes a number of roles that physicians are required to demonstrate to effectively serve the healthcare needs of their patients [3]. The seven CanMEDS roles include medical expert, communicator, collaborator, leader, health advocate, scholar, and professional [3]. A survey evaluating Canadian otolaryngology surgical residents indicated that global health electives may serve as a valuable forum for developing CanMEDS roles [4].

University of Toronto’s (UofT) department of Obstetrics and Gynaecology (ObGyn) has been participating as reproductive health lead in the Academic Model Providing Access to Healthcare (AMPATH) program for over 10 years. AMPATH is a program that partners multiple North American academic institutions with Moi University School of Medicine (MUSOM) and the Moi Teaching and Referral Hospital (MTRH), a tertiary care center in Eldoret, Kenya. AMPATH works in conjunction with the government of Kenya to improve the quality of women’s reproductive health and care. Through this large and multifaceted program, medical students and residents from both UofT and MUSOM have the opportunity to participate in bidirectional ObGyn electives at each other’s setting. UofT trainees have had the opportunity to visit MUSOM/MTRH for over 10 years to obtain global health ObGyn training in a resource-limited setting. Through this elective, trainees experience the realities of acquiring surgical skills in a resource-limiting setting and learn techniques appropriate in these different educational contexts [5]. MTRH has an obstetrical volume of approximately 15,000 deliveries per year, 500 gynecologic surgeries annually, and over 7000 reproductive health-related outpatient visits [5]. The Kenyan setting features conditions of post-abortal care, advanced condylomata, patients with pelvic mass, malaria in pregnancy, pre-eclampsia and eclampsia, and pre-term labor and delivery [5]. The aim of this elective is to provide trainees with an opportunity to develop an interest in Global Women’s Reproductive Health. It includes exposure to issues of cultural sensitivity, health disparities, human rights, and medical ethics [5]. This program therefore provides an opportunity to explore the value of global health electives in furthering and enhancing CanMEDS competencies through trainees’ experiences at a single consistent location.

This study seeks to examine the self-perceived long-term impact that participation in an global health elective has had on CanMEDS competencies and future practice. It was predicted that participation in this elective would generate self-perceived positive impacts on future practice demonstrated through the development and enhancement of the CanMEDS roles.

## Methods

A survey was created through Fluid Survey and was sent to 43 individuals who had participated in the AMPATH global health elective over the past 10 years. Ethics approval was granted by the Health Science Research Ethics Board at UofT. Twenty-three individuals responded (53% response rate) and participated in this mixed method survey evaluating their global health experience in Eldoret, Kenya. Complete data was obtained for 17 of these participants. The survey included 36 quantitative questions; 33 of these were derived from the CanMEDS framework [3] to assess the CanMEDS roles and 3 questions were used to evaluate the frequency of ongoing involvement in global health. The survey also provided the opportunity to qualitatively explain how subjects felt this elective had impacted their practice, elements of their work, or parts of their career.

## Results

Demographic data including age, career status, current practice, and level of education were collected (Table 1). Half of the participants that completed this elective were currently between the ages of 31 and 35, 27% were 25–30, and 22% were between 36 and 40 years of age. The length of elective varied among participants, with 26% completing a 4–6-week elective (the minimum time allowed in our program), 35% completing a 7–9-week elective, and 39% completing a 10–12-week elective. At the time of this study, the majority of participants surveyed worked in obstetrics and gynecology (52%), 13% worked in family practice, 26% practiced in other areas of medicine, and 8% were current medical students.

**Table 1:** Demographic data

Age	25–30	31–35	36–40	> 41
	6 (27%)	11 (50%)	5 (22%)	0
Length of elective	4–6 weeks	7–9 weeks	10–12 weeks	> 3 months
	2 (8%)	11 (47%)	4 (17%)	6 (26%)
Current career status	Medical student	Resident	Fellow	Staff clinician
	2 (8%)	11 (47%)	4 (17%)	6 (26%)
Type of practice now	Academic	Community	Both	Other/N/A
	7 (30%)	1 (4%)	5 (21%)	1 / 9 (4%) / (39%)
Area of medicine	Obstetrics and gynecology	Family	Other	N/A
	12 (52%)	3 (13%)	6 (26%)	2 (8%)

The medical expert, collaborator, leader, scholar, and professional CanMEDS roles were self-perceived to be strongly impacted through this AMPATH global health elective (Table 2). A moderate to major impact was reported among 94% of participants for the medical expert role. For the collaborator role, 76% of participants reported a moderate or major impact on working effectively with physicians and other colleagues. A moderate or major impact was reported among 94% of participants for the leader role, specifically, for the competency of “engaging in stewardship of health care resources” [3]. For the CanMEDS scholar role, 82% of participants reported a moderate or major impact. Lastly, the professional role was reported to have a moderate or major impact among 88% of participants who indicated that participation in this elective contributed to their demonstration of commitment to society.

**Table 2:** Self-perceived impact of global health elective on CanMEDS competencies

CanMEDS roles	Competency	No impact (1) or minor impact (2)	Neutral (3)	Moderate impact (4) or major impact (5)
Medical expert [3]	“Actively contributing, as an individual and as a member of a team providing care, to the continuous improvement of health care quality and patient safety” [3]	0	1 (6%)	16 (94%)
	“Establishing professional therapeutic relationships with patients and their families” [3]	2 (12%)	7 (41%)	8 (47%)
Collaborator [3]	“Working effectively with physicians and other colleagues in the health care profession” [3]	0	4 (24%)	13 (76%)
Leader [3]	“Engaging in stewardship of health care resources” [3]	0	1 (6%)	16 (94%)
	“Responding to an individual patient’s health needs by advocating the with patient within and beyond the clinical environment” [3]	1 (6%)	1 (6%)	16 (94%)
	“Responding to the needs of communities or populations they serve by advocating with them for system-level change in a socially accountable manner” [3]	2 (12%)	1 (6%)	14 (82%)
Scholar [3]	“Contributing to the creating and dissemination of knowledge and practices applicable to health” [3]	2 (12%)	1 (6%)	14 (82%)
Professional [3]	“Demonstrating commitment to society by recognizing and responding to societal expectation in health care” [3]	1 (6%)	1 (6%)	15 (88%)

While a number of positive impacts were perceived among participants, the results also revealed areas that were not perceived as strongly. Lower ratings were found for “planning and performing procedures and therapies for the purpose of assessment and/or management” [3], “documenting and sharing written and electronic information about the medical encounter to optimize clinical decision making, patient safety, confidentiality, and privacy” [3], and “handing over the care of a patient to another health care professional to facilitate continuity of safe patient care” [3].

To assess the impact this elective had on future practice, ongoing involvement in global health activities were surveyed (Table 3). A moderate to significant amount (4 or 5 on a Likert scale) was found in 41% of participants when asked about the frequency of their current involvement with global health work. Additionally, 41% of participants reported a moderate or significant current involvement with teaching trainees about underserved populations locally or abroad. Further, 88% of participants indicated that the AMPATH global health elective was either very influential or extremely influential on their current practice, beliefs, or values.

**Table 3:** Future practice implications

Question	Never (1) or rarely (2)	Occasionally (3)	Moderate amount (4) or a great deal (5)
With what frequency do you have ongoing involvement in global health work?	5 (29%)	5 (29%)	7 (41%)
With what frequency do you have ongoing involvement with teaching trainees about underserved populations locally or abroad?	6 (35%)	4 (24%)	7 (41%)
Question	Not at all (1) or slightly (2)	Somewhat (3)	Very influential (4) or extremely influential (5)
How influential was the AMPATH elective on your current practice, beliefs, or values?	1 (6%)	1 (6%)	15 (88%)

Qualitative data revealed that this elective highlighted innovative ways to deliver primary health care in a low-resource setting and demonstrated how to incorporate global health into a career in medicine. Additional short answer responses highlighted that this global health experience emphasized the role of a physician as a health advocate globally. One participant indicated that “basic clinical knowledge that is acquired and required in medical school can be obtained in classrooms and regular hospital rotations, but a place like Eldoret proves a higher-volume and exposure to more advanced disease processes”. Participants reported that this global health elective was “crucial in shaping their systems perspective,” as it helped shape an interest in innovation in resource-poor contexts, in addition to determining how these innovations might transform our relatively resource-rich contexts. It was also noted that participation was important in learning about privilege and difference.

## Discussion

This study is unique in using the CanMEDS framework to assess the impact of a global health elective on participants. Survey questions were based on the CanMEDS roles and were further divided into competencies specific to each role. Multiple questions were employed, which provided a high level of detail on specific aspects of how the CanMEDS roles relate to the elective, and therefore highlight where potential shortfalls may be. Studies have shown that educators have difficulties translating the CanMEDS framework into their programs [6]. This study therefore provides useful feedback on where program directors may direct their attention to integrate the CanMEDS roles into differing elements of a global health elective.

Although the CanMEDS framework is extensive in defining the necessary competencies to produce good physicians, there may be some limitations with the framework itself. It has been suggested that the framework is valuable in laying the groundwork for postgraduate training, but that it needs to be combined with speciality-specific competencies to ensure that the curriculum aligns with professional practice [6]. Our global health elective used the CanMEDS framework and tailored it to align with obstetrics and gynecology reproductive health outcomes. For example, objectives for Medical Expert included “learning about the common causes of maternal morbidity and mortality in resource poor settings and relevant strategies to reduce such outcomes” [5]. Furthermore, program directors have indicated challenges in evaluating the CanMEDs roles, specifically in evaluating roles other than Medical Expert [7]. Global health electives provide many opportunities to explore the other CanMEDs competencies and evaluate how these roles can be perpetuated and furthered in resource-poor contexts.

Resource-limited contexts provide trainees with a different learning environment than resource-rich contexts [8]. By participating in a resource-poor context, trainees are challenged to maximize and adapt their communication skills and resourcefulness [8]. One study found that international experiences led trainees to rely on clinical findings rather than diagnostic tests [9]. Global health electives also provide trainees with a unique opportunity to become more culturally competent [9]. Additionally, in Kenya, trainees encounter unique clinical circumstances that can challenge their ethical frameworks in a way that is different from what is experienced in Canada [5]. While the context may provide an enriching experience for participants, being competent in a resource-rich context may not translate to competency in a resource-limited setting [8]. However, the positive self-perceived data from this study supports the conclusion that this global health elective provided trainees with a greater appreciation of competency skills as they relate to the reproductive health needs of women in Canada and abroad.

This study was limited due to the nature of survey-based methodologies, where it can be difficult to rate the impact of an experience on a Likert scale. This study also had a low response and completion rate, a chronic concern for survey-based methodologies. Additionally, the study was susceptible to recollection bias as there has been varying amounts of time since trainees participated in this elective, which may have affected each individual’s responses. Furthermore, the sample in this study was biased towards perceiving value in the experience as participants themselves chose to participate in this elective experience. Respondents were therefore likely to be interested in global health and demonstrate a higher inclination to participate in and perceive a positive outcome from global health activities than their non-participating peers.

Future work with this data could include comparing these results to participants who have completed different global health electives in a variety of locations. This will help ascertain the unique impact of the MUSOM/MTRH global health elective. Other future-related research could have participants complete a similar survey immediately after completion of a global health elective. A longitudinal study would also enable a comparison of short-term and long-term outcomes. Other potential uses of this work include identifying the shortfalls for the competencies explored in this study which could in turn inform the creation of stronger learning objectives for electives or other global health medical curricula and better guide trainees in their development of the various CanMEDS roles.

## Conclusion

These findings support our hypothesis that participation in this MUSOM/MTRH GH ObGyn elective would generate self-perceived beneficial impacts on future practice. Global health electives may therefore provide an opportunity for physicians to become more proficient in the CanMEDS roles and also more competent in caring for diverse patient populations.

## Data Availability

The datasets used and/or analyzed for the current study are available from the corresponding author on request.

## Abbreviations

AMPATH: Academic Model Providing Access to Healthcare
MTRH: Moi Teaching and Referral Hospital
MUSOM: Moi University School of Medicine
ObGyn: Obstetrics and Gynaecology
UofT: University of Toronto
